# Supplementary material for: Depletion of HuR in murine skeletal muscle enhances exercise endurance and prevents cancer-induced muscle atrophy
Source: Nat Commun. 2019 Sep 13;10:4171. doi: 10.1038/s41467-019-12186-6 (PMC6744452; doi:10.1038/s41467-019-12186-6)
Supplement: Supplementary file 1 — Supplementary Information [file 41467_2019_12186_MOESM1_ESM.pdf]

Depletion of HuR in murine skeletal muscle enhances exercise endurance and prevents cancer-induced muscle atrophy

Sánchez et al.

Supplementary Information

This file contains Supplementary Figures 1-10 with figure legends, Supplementary Tables 1 and 2 (the primer list) and Information regarding the RNAseq data presented in Supplementary Data 1 (Excel file).

a

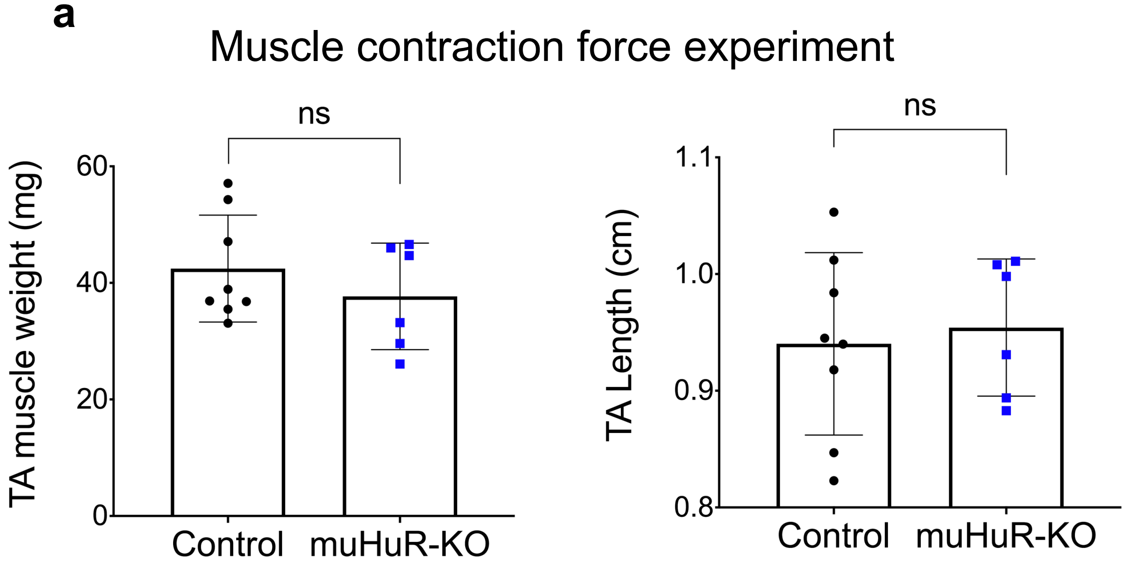

b

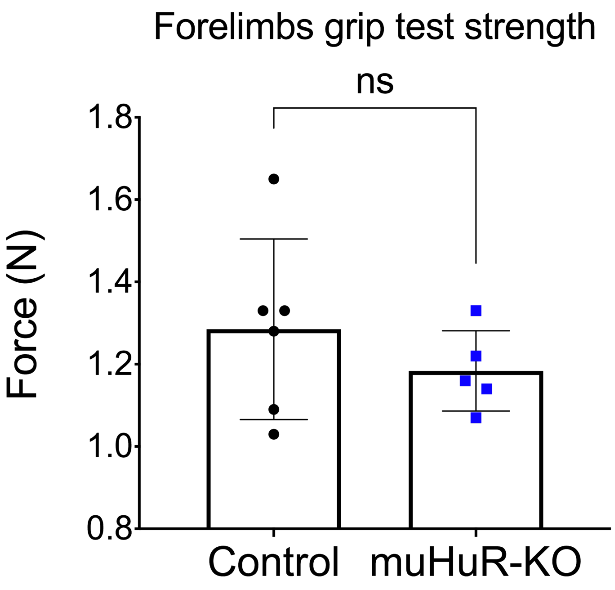

Supplementary Figure 1 HuR muscle specific KO mice exhibit a decrease in muscle contraction strength**. a** Fatigability in Fig. 2a was normalized to TA muscle weight **(Left panel)** and length **(Right panel). b** Grip strength was evaluated on age-matched control and muHuR-KO mice using a digital force gauge. Peak force (N) was measured from forelimbs in 2 sessions. Each session consisted of triplicate measurements taken during 3 consecutive days. 4 days of resting were allowed in between sessions. The results are presented as mean ± S.E.M, unpaired t-test. (**a**, control n=8 and muHuR-KO n=6), (**b**, control n=6 and muHuR-KO n=5). Source Data are provided in the Source Data File.

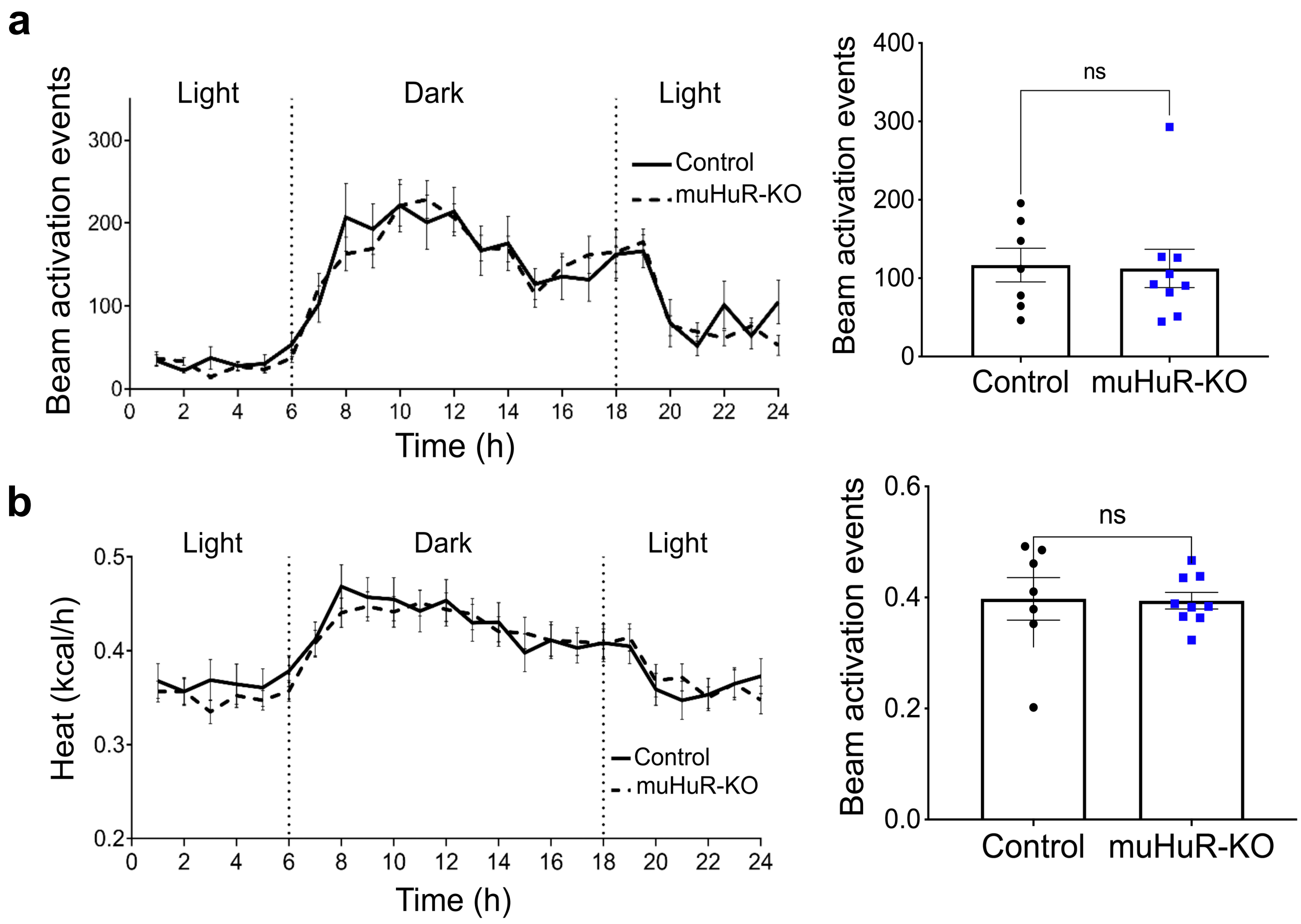

Supplementary Figure 2 muHuR-KO mice display no significant difference in heat production (Energy expenditure) or ambulatory activity when compared to control littermates. **a-b** A Comprehensive Animal Monitoring System (CLAMS; Columbus Instruments, Columbus, OH) was used for a 3-day indirect calorimetry study in age-matched mice under a 12h light–12h dark cycle. (**b**) Heat production was calculated using the following equation: ((3.82 + 1.23 × RER) × VO2). (**a**) (**Left panel**) Ambulatory activity was estimated by the number of infrared beam breaks along the x-axis of the metabolic cage. (**Right panel**) Mean values of beam activation events during 72h in control and muHuR-KO mice. (**b**) (**Left panel**) Graph depicting the average values at each time point. (**Right panel**) Mean values of heat production during 72h in control and muHuR-KO mice. Data was analyzed using CLAMS examination tool (CLAX; Columbus Instruments) version 2.1.0 (**a** and **b** right panels, Control: n=7, muHuR-KO: n=9). The results are presented as mean ± S.E.M, $^*p < 0.05$ unpaired t-test. Source Data are provided in the Source Data File.

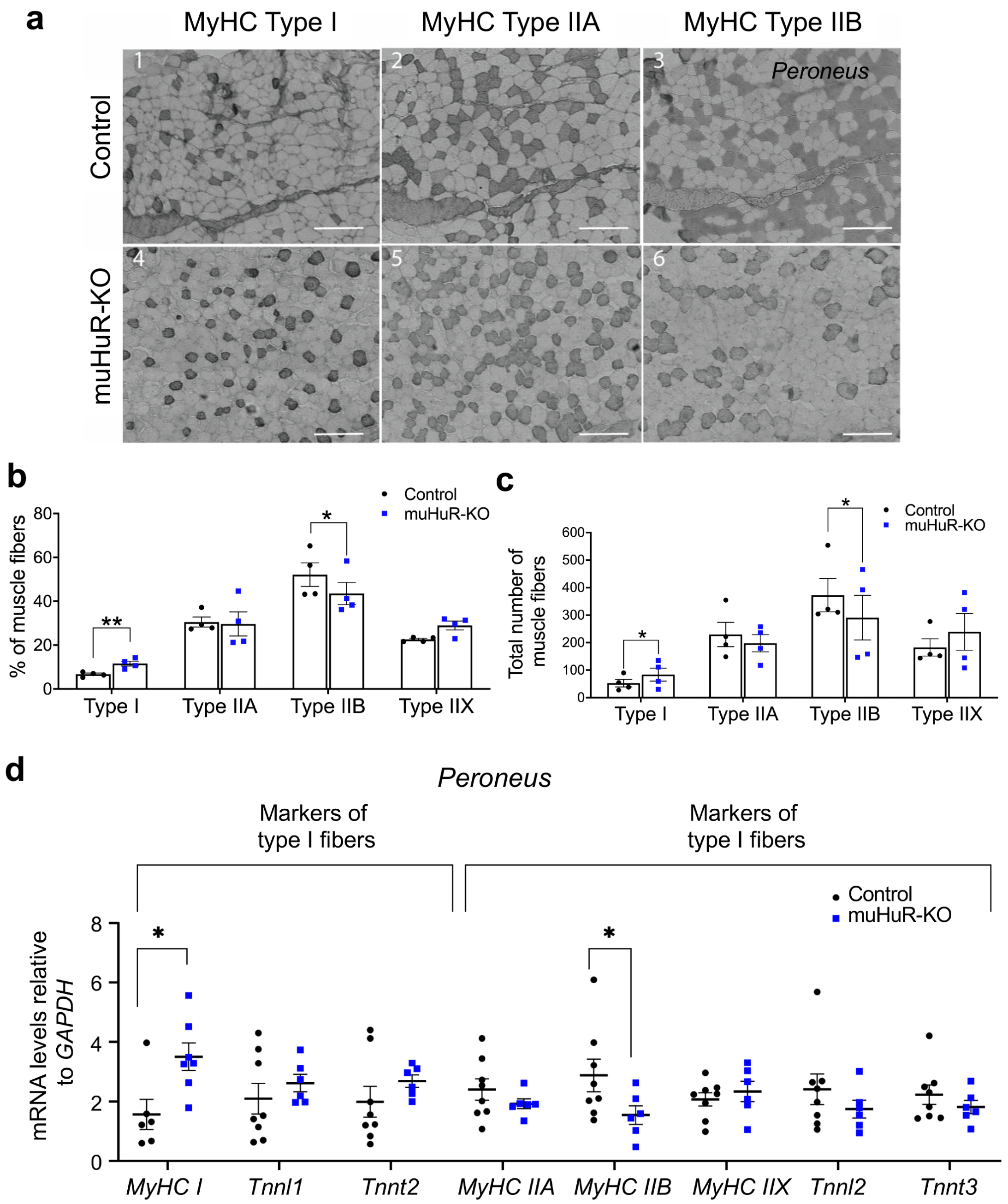

Supplementary Figure 3 Depletion of HuR in skeletal muscle increases the proportion of type I fibers in *peroneus* muscle. **a** Representative photomicrographs of serial sections of *peroneus* muscle from control and muHuR-KO mice taken after immunostaining with anti-Myosin Heavy Chain (MyHC) antibodies type I, type IIA and type IIB. scale bars: 100µm. **b**-**c** Quantification of muscle fibers type I, type IIA, and type IIB, was ascertained manually. Fibers type IIX where calculated by counting the unstained fibers. Results are graphed as the percentage (%) of the total number of fibers per muscle (**b**) and absolute total number of fibers per muscle (**c**) (n=4 mice). **d** mRNA expression of known markers of fiber type specificity (*Tnnl1, Tnnl2, Tnnt2, Tnnt3, MyHC I, MyHC IIA, MyHC IIB, MyHC IIX*) was assessed by RT-qPCR. mRNA levels were standardized against *GAPDH* and plotted relative to the expression in control mice (muHuR-KO n=6 expect for *MyHCI* where n=7), (Control n=8, except for *MyHCI* where n=6)**.** Source Data are provided in the Source Data File.

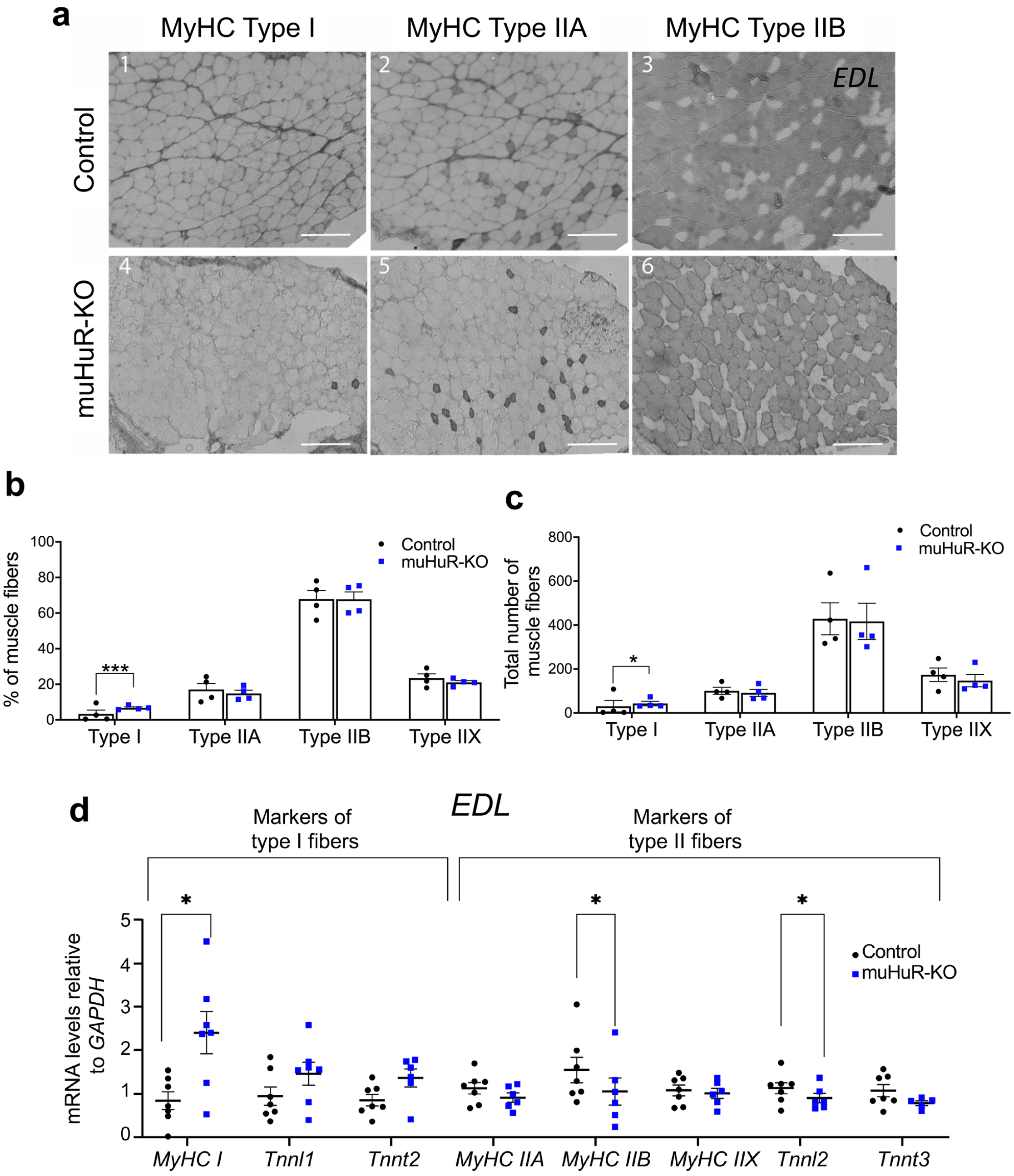

Supplementary Figure 4 Depletion of HuR in skeletal muscle increases the proportion of type I fibers in EDL muscle. **a** Representative photomicrographs of serial sections from of EDL muscle from control and muHuR-KO mice taken after immunostaining with anti-Myosin Heavy Chain (MyHC) antibodies type I, type IIA and type IIB. Scale bars: 100µm. **b-c** Quantification of muscle fibers type I, type IIA, and type IIB, was ascertained manually. Fibers type IIX where calculated by counting the unstained fibers. Results are graphed as percentage (%) of the total number of fibers per muscle (**b**) and total number of fibers per muscle (**c**) (n=4 mice). **d** mRNA expression of known markers of fiber type specificity (*Tnnl1, Tnnl2, Tnnt1, Tnnt3, MyHC I, MyHC IIA, MyHC IIB, MyHC IIX*) was assessed by RT-qPCR. mRNA levels were standardized against *GAPDH* and plotted relative to the expression in control mice. (muHuR-KO n=6 expect for *MyHCI* n=5, *Tnnl1* and *Tnnt2* n=7), (Control n=7, except for *MyHCI* where n=8)**.** Source Data are provided in the Source Data File.

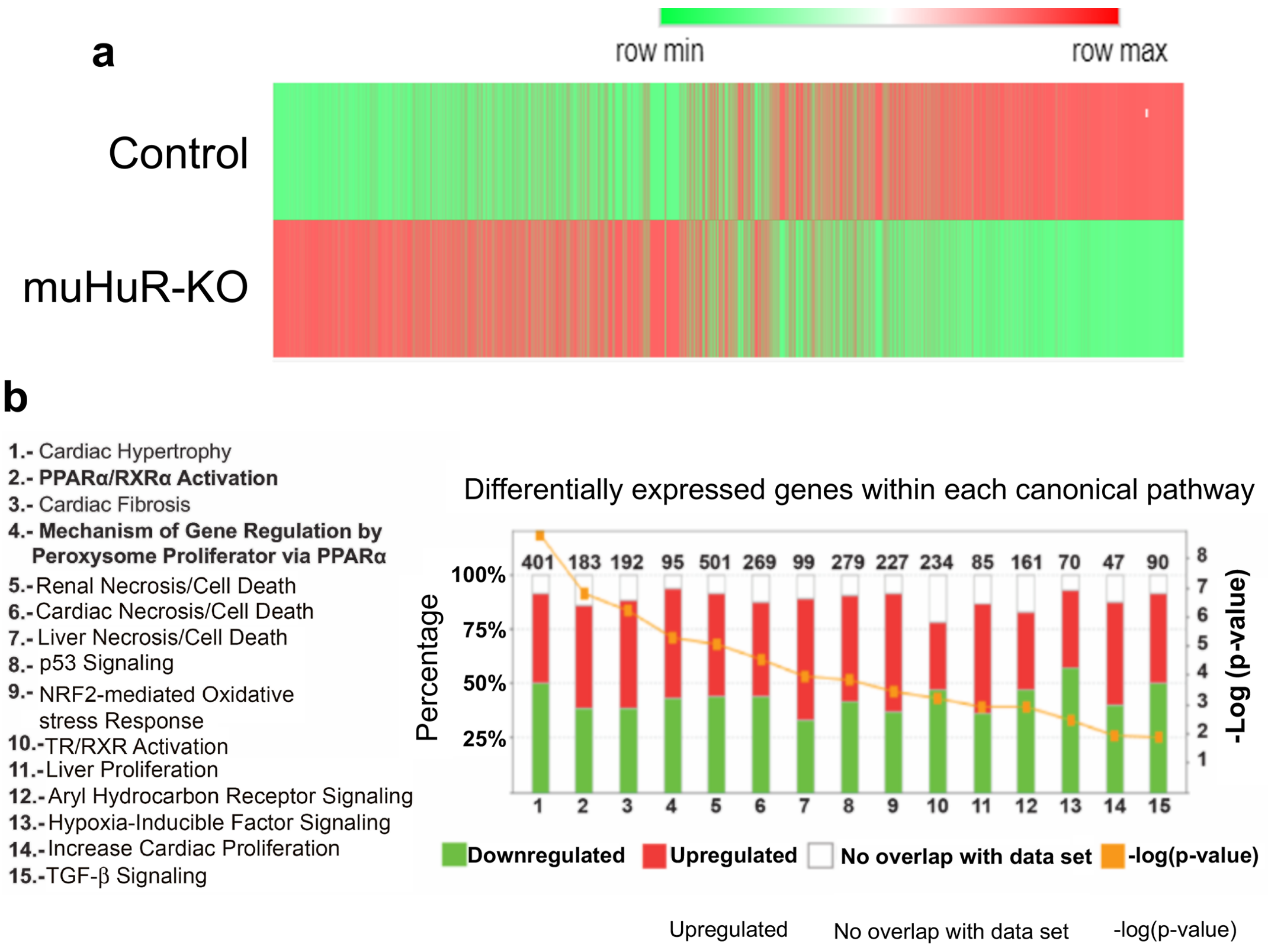

Supplementary Figure 5 Heat Map and IPA analysis of RNAseq data. **a** Heat map analysis of RNA-seq data depicting the changes of gene expression in muHuR-KO mice *soleus* muscles. All transcripts with normalized read counts >0 across all samples were selected for *in silico* analysis and used as input into the website Morpheus to generate a heat map according to the instruction. **b** Differential expression in signaling pathways as analyzed by IPA®. Percentage of genes down-regulated (green), up-regulated (red) or not represented in our data set (white) are shown in the left Y-axis. The total number of genes found within each pathway are shown above each bar graph. The right Y-axis, represented by the orange points, shows the −log *p* value for each pathway. Raw data for RNAseq are provided in Supplementary Table 2.

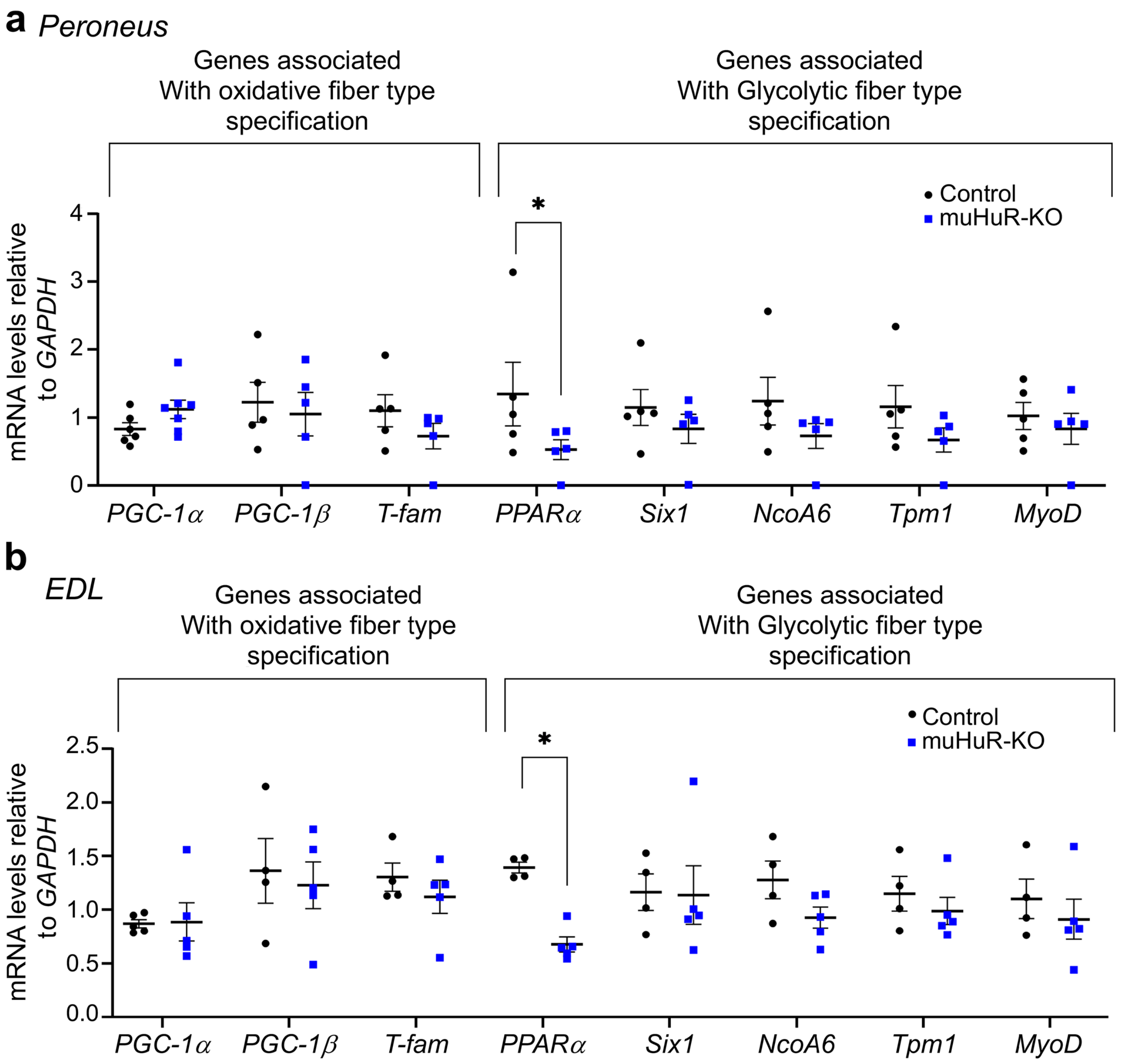

Supplementary Figure 6 HuR differentially affects the expression of mRNAs associated to metabolism in *peroneus* and EDL muscles. **a**-**b** Total RNA was isolated from *peroneus* (**a**) or EDL muscles (**b**) of control and muHuR-KO mice and relative expression level of genes associated to PPAR signaling and/or fiber type specification (*PGC-1α, PGC-1β, Tfam, PPARα, Six1, Tpm1, NCOA6, MyoD*) was assessed by RT-qPCR. Relative mRNA levels were standardized against *GAPDH* and plotted relatively to the expression in control mice (muHuR-KO n=6, (Control n=4, except for *PGC-1α* where n=5). The results are presented as mean ± S.E.M, *p < 0.05, **p < 0.005 unpaired t-test. Source Data are provided in the Source Data File.

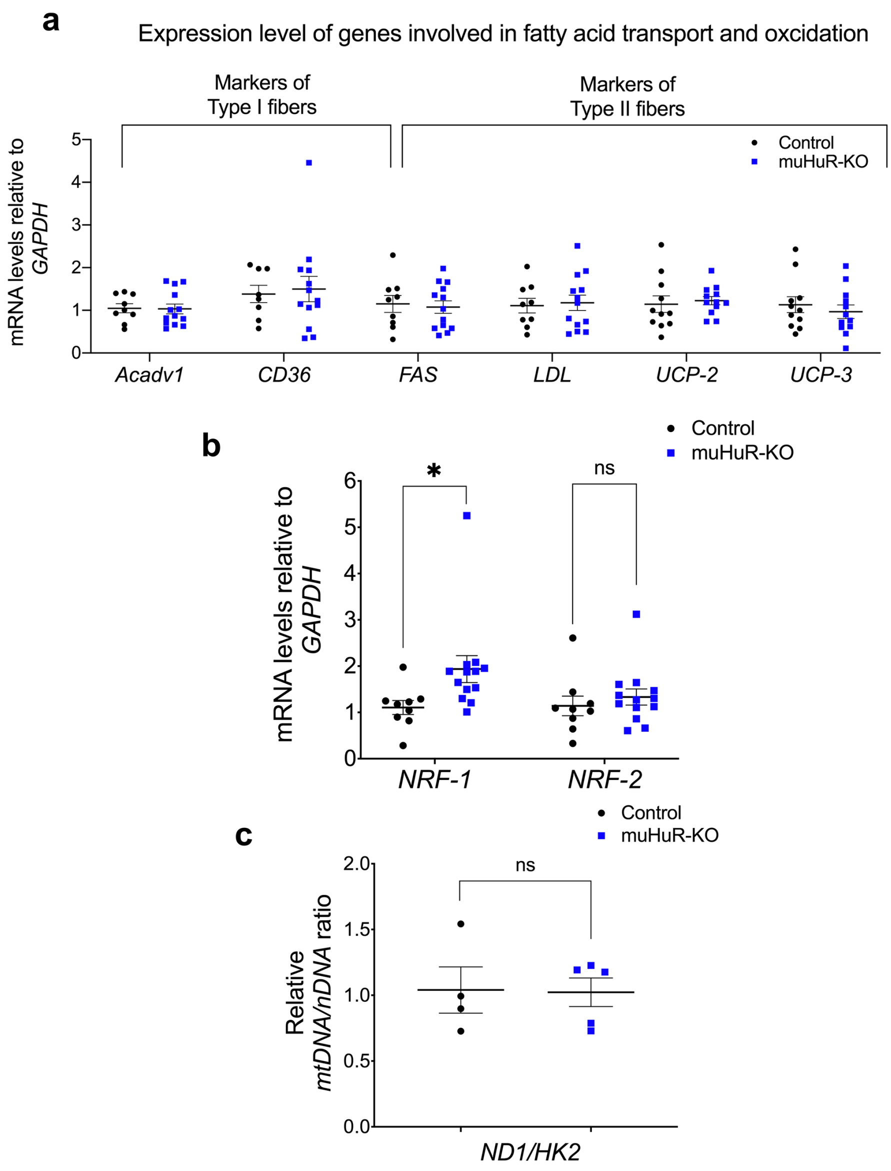

Supplementary Figure 7 Effect of HuR depletion in lipid metabolism and oxidative phosphorylation. **a**-**b** Total RNA was isolated from *soleus* muscles from control and muHuR-KO mice and relative expression of genes involved in (**a**) fatty acid transporters and oxidation [*Acadv1* (control n=9, muHuR-KO n=12)*, CD36* (control n=8, muHuR-KO n=13)*, FAS* (control n=9, muHuR-KO n=13)*, LDL* (control n=9, muHuR-KO n=12)*, UCP-2* and *UCP-3* (control n=11, muHuR-KO n=13] and (**b**) mitochondrial biogenesis [*NRF-1, NRF-2* (control n=9, muHuR-KO n=13)] was assessed by RT-qPCR in *soleus* muscles of Ctl and muHuR-KO mice. mRNA levels were standardized against *GAPDH* and plotted relatively to control animals. **c** DNA extracted from *gastrocnemius* muscle was used to determine the mtDNA/nDNA ratio. Expression levels were standardized against Hexokinase 2 (HK2) and plotted relatively to control animals (control n=4, muHuR-KO n=5). The results are presented as mean ± S.E.M, *p < 0.05 unpaired t-test. Source Data are provided in the Source Data File.

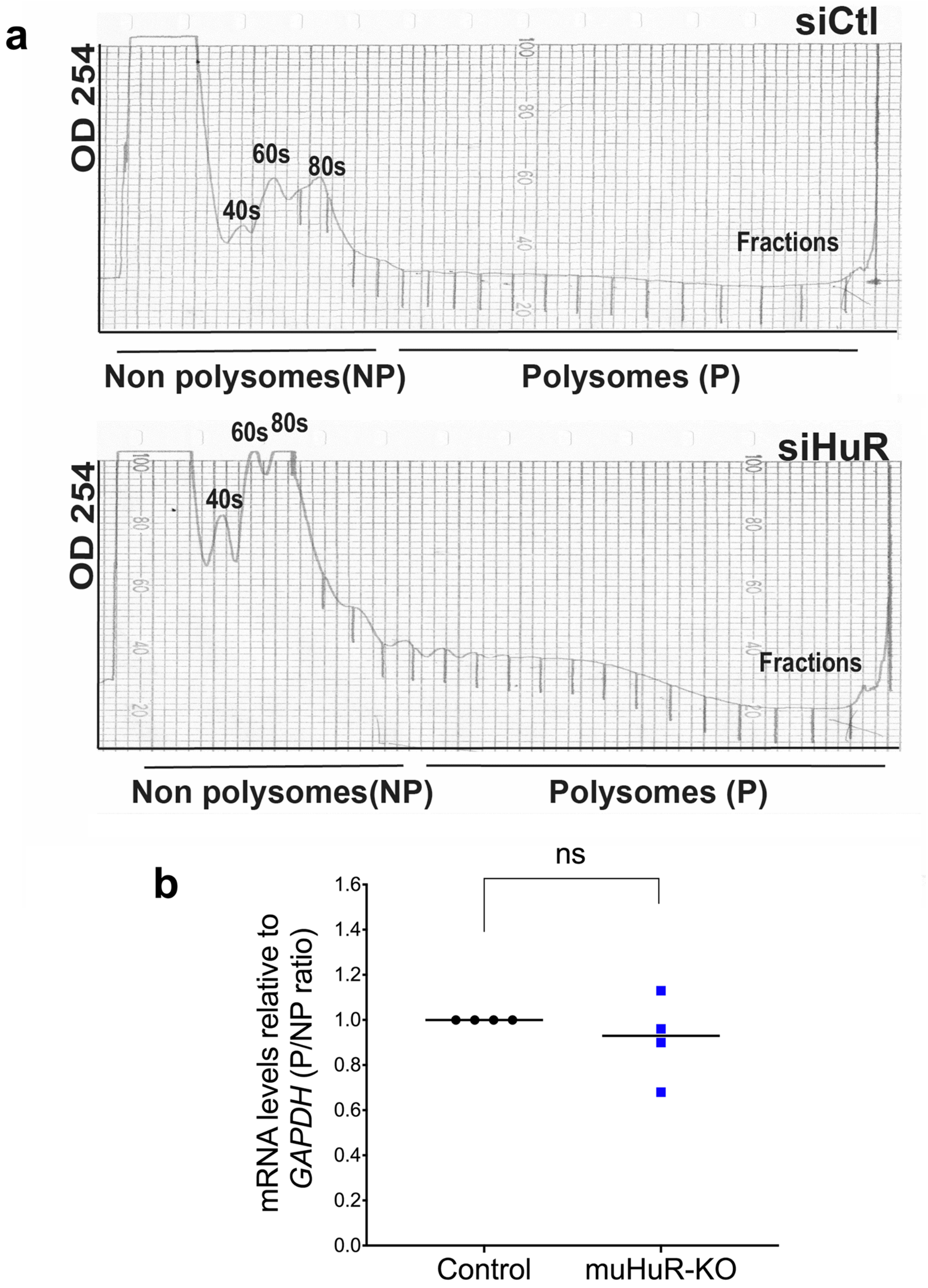

Supplementary Figure 8 HuR does not regulate the translation of PGC-1α mRNA. Cytoplasmic extracts obtained from C2C12 myoblasts treated with or without siHuR were prepared and fractionated on sucrose gradients (15–50% w/v). **a** Fractions were divided into two groups; non-polysome (NP, fractions 1–6) and polysome (P, fractions 7–20). **b** The level of *PGC-1α* and *GAPDH* mRNAs in the Polysome and Non-Polysome were quantified by RT–qPCR using the ΔΔCt method and plotted as a Polysome to Non-Polysome ratio (n=4). The results are presented as mean ± S.E.M, *$p < 0.05$ unpaired t-test. Source Data are provided in the Source Data File.

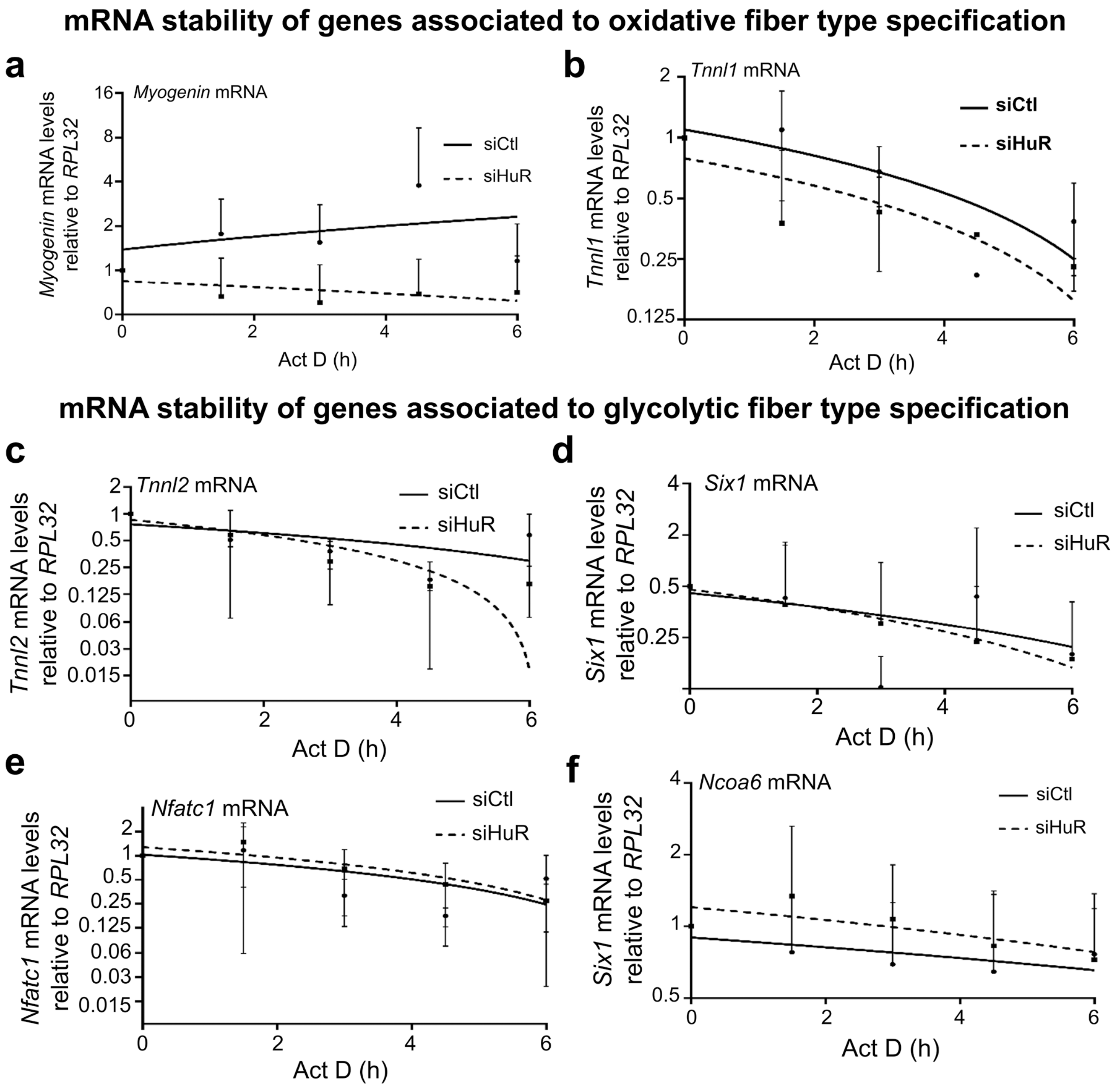

Supplementary Figure 9 HuR differentially affects the stability of mRNAs associated to fiber type specification. **a-f** C2C12 myoblasts treated with or without siHuR were used to assess the stability of *Myogenin* (**a**), *Tnnl1* (**b**), *Tnnl2* (**c**), *Six1* (**d**), *NFATc1* (**e**) *and NCOA6* (**f**) mRNAs. Cells were treated with actinomycin D (ActD) for 0, 1.5, 3, 4.5 or 6 hours and mRNA from the different time points was process by RT-qPCR. mRNA levels of the genes of interest was standardized against *RPL32* mRNA levels and plotted as a percent of the abundance of mRNA at time 0 of ActD treatment, which is considered as 1 (n=3, except for Tnnl1 where n=2). The line of best fit was determined by linear regression using the data points for siCtl and siHuR. Error bars represent ± S.E.M. Source Data are provided in the Source Data File.

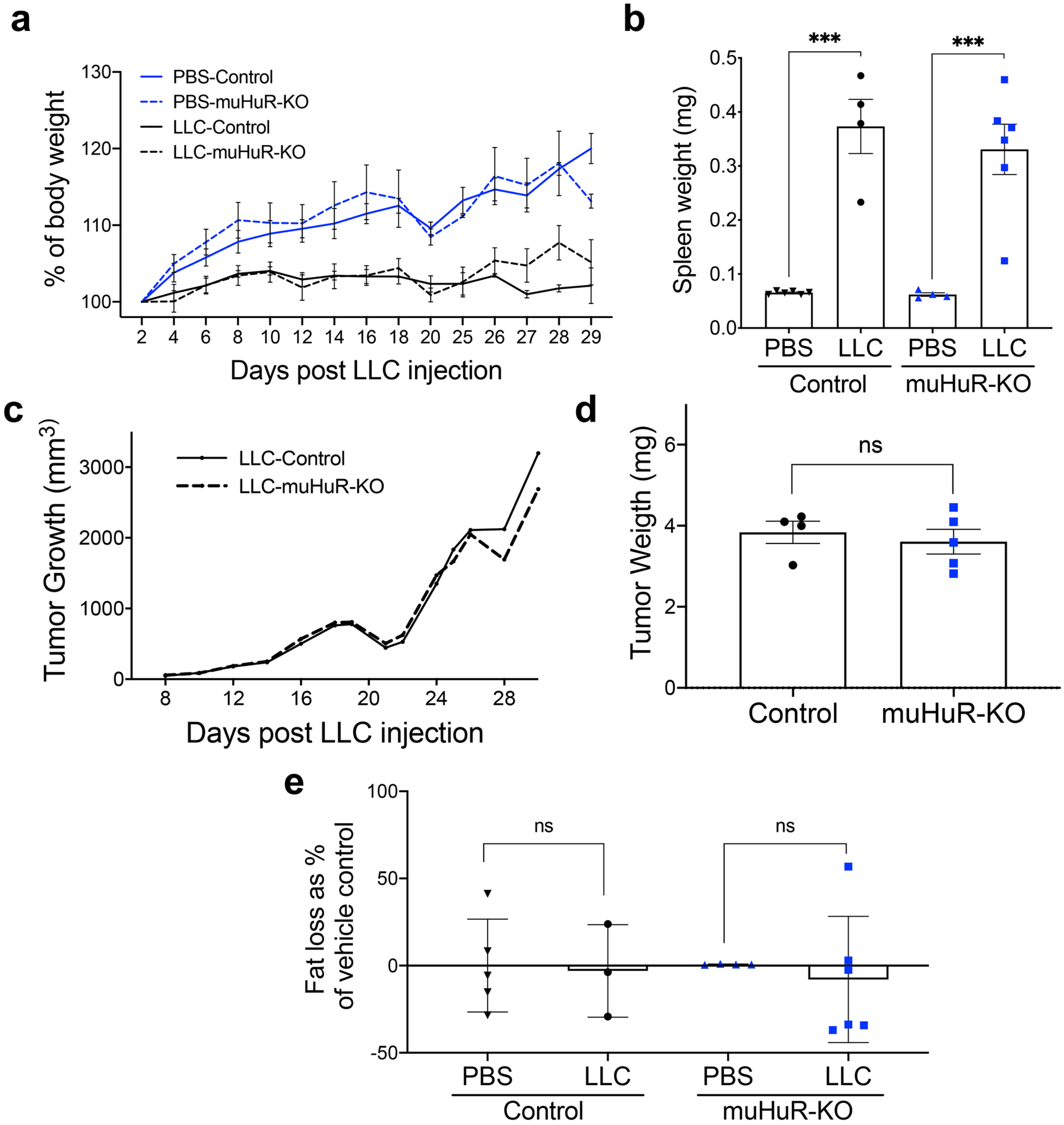

Supplementary Figure 10 Validation of the LLC model in control and muHuR-KO mice. **a**-**e** Control and muHuR-KO mice were inoculated subcutaneously in their right flank with LLC cells or PBS and evaluated 29 days after inoculation by measuring (**a**) total body weight gain (LLC-Control n=4, LLC-muHuR-KO n=5). (n=5 LLC-Control n=4), (**b**) sign of inflammation (spleen weight) (LLC-Control and PBS-muHuR n=4, PBS-Control and PBS-Control and LLC-muHuR-KO n=6), (**c**) tumor growth progression (n=5 except for LLC-Control where n=4), (**d**) tumor burden (LLC-Control n=4, and LLC-muHuR-KO n=5), (**e**) hindlimb fat pad loss (LLC-Control and PBS-muHuR n=4, PBS-Control n=5 and LLC-muHuR-KO n=6). (**a**-**e**). The results are presented as mean ± S.E.M. Source Data are provided in the Source Data File.

	Total number of fibers counted				
	Muscle	MyHC Type I	MyHC Type IIA	MyHC Type IIB	MyHC Type IIX
Control	EDL	1.53 ±0.50	22.66 ±2.26	75.81 ±2.60	22.97 ±1.95
	PER	5.84 ±0.51	33.31 ±1.87	60.85 ±2.30	23.42 ±1.79
	SOL	43.12 ±1.43	52.81 ±1.65	0.46 ±0.20	8.22 ±2.18
muHuR-OK	EDL	7.31 ±0.95***	17.81 ±1.67	74.88 ±2.38	21.01 ±1.22
	PER	12.76 ±1.99**	39.14 ±4.08	48.10 ±4.76*	26.65 ±3.29
	SOL	60.23 ±2.04***	36.75 ±1.59***	0.00 ±0.00*	15.08 ±1.62

Abbreviations: EDL (Extensor digitorum longus), PER (Peroneus), SOL (Soleus). Results are shown as mean ± S.E.M. *$p < 0.05$, **$p < 0.005$, ***$p < 0.001$

Supplementary Table 1 Effect of HuR ablation on fiber type composition in *soleus* (SOL), EDL and *peroneus* (PER) muscles.

Supplementary Table 2 Sequence of primers and siRNAs use in this study

Methodology	Targeted Gene	Sequence
PCR	LoxP	Forward 5'-TGG TTA TGA AGA CCA CAT GGC GGA AGA-3'<br>Reverse 5'-AGC TTA GCA GGT ACC GTC TCC-3'.
	Cre	Forward 5'-CAT TTG GGC CAG CTA AAC AT-3'<br>Reverse 5'-CGG ATC ATC AGC TAC ACC AG-3'.
	HuR exon 2	Forward 5'-ATA TCA TGT TCC CAA CTC CC-3'<br>Reverse 5'-TGG CAC TCA CTG AAC TGG AA-3'.
siRNA	HuR	Sense: 5'- CAAACTCAGGAGCTTCTTTTTTGTTTATCATAAT-3'<br>Anti-sense: 5'- ATTATGATAAACAAAAAAGAAGCTCCTGAGTTTG -3'
	CTL	Sense: 5'- TGTGTATTGTTTATTGTTTTGTGTGTTGTTTGTAAA -3'<br>Anti-sense: 5'- TGTGTATTGTTTATTGTTTTGTGTGTTGTTTGTAAA -3'
	KSRP	Sense: 5'- CAAACTCAGGAGCTTCTTTTTTGTTTATCATAAT-3'<br>Anti-sense: 5'- ATTATGATAAACAAAAAAGAAGCTCCTGAGTTTG -3'
qPCR	Tnnl1	Forward 5'-GAA CAC GAG GAG CGA GAG G-3'<br>Reverse 5'-CCT TCA GCT TCA GGT CCT TG-3'.
	Tnnl2	Forward 5'-GGA GGG TGC GTA TGT CTG C-3'<br>Reverse 5'-GGG AAG TGG GCA GTT AGG AC-3'.
	Tnnt1	Forward 5'-GCC CAG GAG CTG TCA GAA T-3'<br>Reverse 5'-CTC CAC ACA GCA GGT CAT GT-3'.
	Tnnt3	Forward 5'-TGA TAT CAC CAC CCT CAG GA-3'<br>Reverse 5'-TCC TGA GTT CCC AAA GAT GC-3'.
	MyHC I	Forward 5'-CTC AAG CTG CTC AGC AAT CTA TTT-3'<br>Reverse 5'-GGA GCG CAA GTTTGT CAT AAG T -3'.
	MyHC IIA	Forward 5′-AGG CGG CTG AGG AGC ACG TA-3′<br>Reverse 5′-GCG GCA CAA GCA GCG TTG G-3′.
	MyHC IIX	Forward 5'-GAG GGA CAG TTC ATC GAT AGC AA-3'<br>Reverse 5'-GGG CCA ACT TGT CAT CTC TCA T -3'.
	MyHC IIB	Forward 5′-CAC CTG GAC GAT GCT CTC AGA-3′<br>Reverse 5′-GCT CTT GCT CGG CCA CTC T-3′.
	Tfam	Forward 5'-CCA AAA AGA CCT CGT TCA GC-3'<br>Reverse 5'-CCA TCT GCT CTT CCC AAG AC-3'
	PGC-1α	Forward 5′-CAG GAA CAG CAG CAG AGA CA-3′<br>Reverse 5′-GTT AGG CCT GCA GTT CCA GA-3′.
	PGC-1β	Forward 5'-GCC AGA AGC ACG GTT TTA TC-3'<br>Reverse 5'-ATC CAT GGC TTC GTA CTT GC-3'.
	Six 1	Forward 5'-AGG GAG AAA CGG GAG CTG-3'<br>Reverse 5'-GGG GGT GAG AAC TCC TCT TC-3'.
	NCOA6	Forward 5'-CCA TAG CCT CTG GAC AAA GC-3'<br>Reverse 5'-TGG ATT TTC GCT TGG AT-3'.
	NFATc1	Forward 5'-TGG AGA AGC AGA GCA CAG AC-3'<br>Reverse 5'-GCG GAA AGG TGG TAT CTC AA-3'.
	Atrogin 1	Forward 5'-GAC CGG CTA CTG TGG AAG AG-3'<br>Reverse 5'-CCA GGA GAG AAT GTG GCA GT-3'

	Murf1	Forward 5'-GAG CAA GGC TTT GAG AAC ATG GAC T-3'<br>Reverse 5'-GCG TCC AGA GCG TGT CTC ACT-3'.
	TPM1	Forward 5'-TGC TTT TCT CCA ATT TGG TT-3'.<br>Reverse 5'-GGG CTG AGC TCT CAG AAG G-3'
	RPL32	Forward 5'-TTC TTC CTC GGC GCT GCC TAC GA-3'<br>Reverse 5'-AAC CTT CTC CGC ACC CTG TTG TCA-3'.
	GAPDH	Forward 5'-AAG GTC ATC CCA GAG CTG AA-3'<br>Reverse 5'-AGG AGA CAA CCT GGT CCT CA-3'.
	NRF-1	Forward 5'-CAGCACCTTTGGAGAATGTG-3'<br>Reverse 5'-CCTGGGTCATTTTGTCCACA-3'.
	NRF-2	Forward 5'-GATCCGCCAGCTACTCCCAGGTTG-3'<br>Reverse 5'-CAGGGCAAGCGACTCATGGTCATC-3'.
	MyoD	Forward 5'-CGACACCGCCTACTACAGTG-3'<br>Reverse 5'-TTCTGTGTCGCTTAGGGATG-3'
	Myogenin	Forward 5'-CTACAGGCCTTGCTCAGCTC-3'<br>Reverse 5'-AGATTGTGGGCGTCTGTAGG-3'
	UCP-2	Forward 5'-TCTACAATGGGCTGGTCGC-3'<br>Reverse 5'-CAAGCGGAGAAAGGAAGGC-3'.
	UCP-3	Forward 5'-CCTACAGAACCATCGCCAGG-3'<br>Reverse 5'-ACCGGGGAGGCCACCACTGT-3'.
	Acadv1	Forward 5'-GGAGGACGACACTTTGCAGG-3'<br>Reverse 5'-AGCGAGCATACTGGGTATTAGA-3'.
	CD36	Forward 5'-GATGACGTGGCAAAGAACAG-3'<br>Reverse 5'-TCCTCGGGGTCCTGAGTTAT-3'.
	FAS	Forward 5'-AGAGATCCCGAGACGCTTCT-3'<br>Reverse 5'-GCCTGGTAGGCATTCTGTAGT-3'.
	LDL	Forward 5'-TGTGAATTTGGTGGCTGAAAAC-3'<br>Reverse 5'-AATAGGGAAGAAGATGGACAGGAAC-3'.
	ND1	Forward 5'-CTAGCAGAAACAAACCGGGC-3'<br>Reverse 5'-CCGGCTGCGTATTCTACGTT-3'.
	HK2	Forward 5'-GCCAGCCTCTCCTGATTTTAGTGT-3'<br>Reverse 5'-GGGAACACAAAAGACCTCTTCTGG-3'.
	PPARα	Forward 5'-GCGTACGGCAATGGCTTTAT-3'<br>Reverse 5'-ACAGAACGGCTTCCTCAGGTT-3'.

Supplementary Data 1 List of differentially expressed genes as analyzed by RNA-Seq in the *soleus* muscle from muHuR-KO and control mice (log2 FC > 0.5 or < −0.5, p=0.05) (See attached Excel File). The raw RNASeq data have been deposited into NCBI Gene Expression Omnibus (GEO) data base under accession number GSE134241.
