# Supplementary material for: Depletion of HuR in murine skeletal muscle enhances exercise endurance and prevents cancer-induced muscle atrophy
Source: Nat Commun. 2019 Sep 13;10:4171. doi: 10.1038/s41467-019-12186-6 (PMC6744452; doi:10.1038/s41467-019-12186-6)
Supplement: Supplementary file 4 — Description of Additional Supplementary Files [file 41467_2019_12186_MOESM4_ESM.docx]

**Title:** Supplementary Data 1
**Description:** List of differentially expressed genes as analyzed by RNA-Seq in the soleus muscle from muHuR-KO and control mice (log2 FC > 0.5 or < −0.5, p=0.05) (See attached Excel File). The raw RNASeq data have been deposited into NCBI Gene Expression Omnibus (GEO) data base under accession number GSE134241.
